# Effects of *In Ovo* Injection of Butyrate Glyceride Blend on Broiler Chicks’ Performance

**DOI:** 10.3390/ani16152293

**Published:** 2026-07-23

**Authors:** Gehad Mohamed Fawaz, Mohamed Ibrahim Hassan, Karim Mohamed El-Sabrout, Ahmed Mohamed Abd El-Hady, Assem Mohamed Safwat

**Affiliations:** 1 Poultry Production Department, Faculty of Agriculture (El-Shatby), Alexandria University, Alexandria 21545, Egypt; gehadfwaz10@gmail.com (G.M.F.); ahmed75atta@yahoo.com (A.M.A.E.-H.); 2 Livestock Research Department, Arid Lands Cultivation Research Institute, City of Scientific Research and Technological Applications (SRTA-City), New Borg El-Arab, Alexandria 21934, Egypt; mibrahem1@yahoo.com

**Keywords:** antioxidant, body weight, blood biochemical parameters, broiler chickens, butyric acid, carcass traits, chick quality, hatchability characteristics, immunity, *in ovo* nutrition, intestinal microbiota

## Abstract

*In ovo* supplementation of bioactive substances represents an effective nutritional intervention for enhancing broiler development from the embryonic stage through market age. This study investigates the impact of injecting graded levels of *in ovo* BGB on the physiological and developmental trajectories of broiler chicks. The investigation spans from embryonic development to a 35-day post-hatch period, assessing hatchability characteristics, chick quality, physiological indices, and growth performance. Based on the results, BGB improved chick quality at hatch by increasing body weight (*p* = 0.0484), Pasgar score (*p* = 0.0296), and several blood biochemical indicators, such as serum protein (*p* = 0.0005) and thyroid hormone concentrations (T3, *p* = 0.0001), while reducing blood glucose levels (*p* = 0.0180). Most notably, BGB promoted substantial improvements in intestinal morphology, including greater villus height (*p* = 0.0001), villus surface area (*p* = 0.0001), and villus-to-crypt ratio (*p* = 0.0020), indicating enhanced digestive and absorptive capacity. These physiological and intestinal benefits translated into superior post-hatch growth performance, characterized by higher body weight gain (*p* = 0.0001) and improved feed conversion efficiency (*p* = 0.0001). Notably, dressing percentage was significantly affected by a quadratic response (*p* = 0.0448), where the 0.30% BGB concentration yielded the highest dressing yield (81.1%). Furthermore, humoral immunity was strongly enhanced; immunoglobulin G (IgG) levels demonstrated a highly significant quadratic response (*p* = 0.0004), maximizing at 11.23 mg/mL in the 0.30% BGB group. Overall, the findings demonstrate that *in ovo* BGB administration, particularly at the 0.30% level, acts as a powerful developmental programming strategy that supports intestinal maturation, metabolic function, immune response, carcass traits, and long-term growth performance in broiler chickens.

## Simple Summary

Early nutritional interventions during embryonic development can influence the health and productivity of broiler chickens after hatch. This study demonstrated that *in ovo* supplementation of butyrate glyceride blend (BGB) improved chick quality, enhanced blood metabolic profiles, and promoted intestinal development without negatively affecting hatchability. Birds receiving BGB showed better intestinal morphology, increased concentrations of key physiological indicators, including immunological and antioxidant parameters, and improved growth performance and feed utilization during the fattening period. The most favorable responses were generally observed at the 0.30% supplementation level. These findings suggest that *in ovo* BGB administration can be used as an effective nutritional tool to support broiler health and production efficiency.

## 1. Introduction

The use of *in ovo* injection technology has emerged as a sophisticated method to enhance the embryonic development and post-hatch performance of broiler chickens [1,2,3]. By delivering bioactive compounds directly into the amniotic fluid or yolk sac during embryogenesis, researchers can bypass the limitations of early-life fasting and jumpstart intestinal maturation [1,2]. Butyric acid and butyrin have gained considerable attention in poultry nutrition due to their important role in maintaining intestinal health and development [3,4,5]. As a major energy source for intestinal epithelial cells, butyrate glycerides support enterocyte proliferation, enhance gut barrier integrity, and contribute to the establishment of a favorable intestinal environment during embryonic development. Furthermore, its antimicrobial and immunomodulatory properties may help shape the early gut microbial ecosystem, thereby improving nutrient utilization and promoting post-hatch growth and health [5,6].

Early gut health is a primary determinant of growth efficiency in modern broiler strains [1]. A butyrate glyceride blend (BGB) acts as an effective dietary source of short-chain fatty acids (SCFAs), known for enhancing gut health in poultry [3,7]. Research indicates that providing these esterified nutrients *in ovo* can mitigate the “post-hatch dip” in growth by ensuring that the gastrointestinal tract is functionally prepared for exogenous feed [1,8]. Furthermore, organic acid derivatives structured as a butyrate glyceride blend enhance the expression of tight junction proteins, thereby strengthening the mucosal barrier and improving nutrient absorption kinetics [3,9,10,11]. Beyond structural development, the introduction of butyric acid and butyrate glycerides into the embryonic environment triggers positive shifts in the chick’s immune profile and antioxidant status [1,5,12,13]. It serves as a signaling molecule that can downregulate pro-inflammatory cytokines while promoting the colonization of beneficial lactic acid bacteria [1,5,11,13]. It also allows the bird to allocate more metabolic energy toward muscle deposition [1,13]. It has been shown to improve growth performance and carcass traits while inhibiting the growth of pathogenic bacteria [2,4,5,10]. Administering these substances via *in ovo* delivery ensures that every embryo receives a uniform dose of the trophic agent, leading to more homogeneous flock performance [8,14].

While current research establishes that *in ovo* delivery of butyrate derivatives effectively jumpstarts intestinal maturation and supports early development, several critical knowledge gaps remain. Most existing studies focus primarily on short-term gut morphology or broad growth metrics, leaving a definitive lack of clarity regarding how different graded doses of a BGB systematically alter long-term physiological trajectories up to market age. This study is necessary to resolve these ambiguities by providing a comprehensive, holistic evaluation of BGB injection levels. It aims to investigate the effects of *in ovo* injection of BGB at various doses on the developmental and physiological responses of broiler chicks. The evaluation includes hatchability traits and early chick quality, post-hatch growth performance over a 35-day rearing period, carcass characteristics, morphological and histological assessments of vital organs, intestinal microbiota count, as well as immunological and antioxidant indices.

## 2. Materials and Methods

### 2.1. Experimental Site and Ethical Approval

The present study was conducted at Lion Hatchery, a facility affiliated with the National Company, located in the Nubaria region, Egypt. All experimental procedures were performed in accordance with institutional guidelines for the care and use of animals in research. The experimental protocol was approved by the Institutional Animal Care and Use Committee (IACUC) of Alexandria University, Egypt (approval No.: Alex. Agri. 092506116).

### 2.2. Experimental Design and Egg Management

A total of 500 fertile eggs were obtained on the day of setting from 49-week-old Cobb 500 broiler breeder flocks. Eggs were individually weighed prior to incubation to ensure uniformity.

Fertility was assessed by candling on day 10 of incubation, and infertile or non-viable eggs were removed and replaced with viable eggs of similar weight to maintain a consistent mean egg weight across treatments. Following fertility confirmation, eggs were randomly allocated into five experimental groups, each comprising five replicates of 20 eggs. All groups were standardized for egg weight at setting (average: 69.51 ± 0.24 g).

The experimental treatments were as follows:

T1: Negative control (non-injected eggs).
T2: Positive control (eggs injected with sterile saline).
T3: Eggs injected with 0.15% BGB solution.
T4: Eggs injected with 0.30% BGB solution.
T5: Eggs injected with 0.45% BGB solution.

### 2.3. Preparation of Bgb Solutions

The BGB used in this study was a commercial product (Monobutyrin^®^, Hubei Horwath Biotechnology Co., Ltd., Xianning, China), supplied by Forte Pharma Animal Health Products (Egypt). According to the manufacturer’s specifications, each liter contained 450 g mono butyrin, 330 g di butyrin, 60 g tri butyrin, 10 g free butyric acid, and 150 g glycerol (carrier). The butyric acid purity of the glyceride esters was 54.33%, 75.86%, and 87.41% for mono butyrin, di butyrin, and tri butyrin, respectively.

BGB solutions were prepared on a volume/volume (*v*/*v*) basis at concentrations of 0.15, 0.30, and 0.45% using sterile physiological saline (0.9% NaCl) as the diluent.

Briefly, 0.15 mL, 0.30 mL, and 0.45 mL of BGB were each diluted with sterile saline to a final volume of 100 mL to obtain the respective concentrations. All solutions were freshly prepared under aseptic conditions immediately prior to *in ovo* injection and thoroughly mixed to ensure homogeneity.

### 2.4. Incubation Conditions and In Ovo Injection Procedure

Eggs were incubated in a Petersime multi-stage incubator (Petersime NV, Zulte, Belgium, Belgium) under standard conditions (99.5 °F and 55% wet-bulb humidity). Automatic turning was performed 24 times daily at a 45° angle until day 18 of incubation. On day 18, eggs were candled to confirm embryonic viability, and only viable eggs were transferred to the hatcher. During the injection procedure, eggs were removed from the incubator for approximately 20 min per tray to maintain uniform handling conditions.

A small opening (approximately 1–2 mm in diameter) was created at the broad end of each egg, directly above the air cell, using a sterile mini grinder. The shell surface was disinfected with 70% ethanol before drilling to maintain aseptic conditions. Eggs assigned to treatments T2–T5 were injected with 0.30 mL of their respective solutions into the amniotic cavity using a sterile 21-gauge needle. The needle was inserted vertically through the air cell to a depth of approximately 25 mm until the amniotic cavity was reached. The injection site and depth were selected according to standard *in ovo* injection procedures to ensure deposition of the solution into the amniotic fluid. A fresh, sterile needle and syringe were used for each treatment to minimize the risk of cross-contamination. After the injection, the needle was carefully withdrawn, and the opening was immediately sealed with sterile melted paraffin wax. All eggs, including the uninjected control group, were then returned to the hatcher and incubated under standard hatching conditions (98.5 °F and 75% relative humidity) until hatch.

### 2.5. Hatchability and Post-Hatch Measurements

#### 2.5.1. Hatching Traits

At hatch (day 21), all chicks were counted and individually weighed. The following parameters were calculated:

Chick yield (%) = (Chick weight at hatch/Initial egg weight) × 100

Hatchability of fertile eggs (%) = (Number of hatched chicks/Number of fertile eggs) × 100

#### 2.5.2. Chick Quality Assessment

Ten chicks per replicate were randomly selected for quality evaluation. Chick length (cm) and shank length (cm) were also recorded as morphometric parameters. Chick quality was further assessed using the Pasgar scoring system, based on five criteria: reflex, navel condition, hocks/legs, beak, and abdomen. Each chick was assigned a score out of 10, with one point deducted for each observed defect. Average scores were calculated per treatment, with values ≥9 indicating high-quality chicks.

#### 2.5.3. Relative Organ Weights

At 1 day of age, a sample of 10 birds/treatment was selected for slaughter traits. Chicks were individually weighed and humanely slaughtered. After bleeding and evisceration, the liver, heart, gizzard, spleen, intestine, and bursa of Fabricius were excised and weighed individually. Relative organ weights were expressed as a percentage of pre-slaughter body weight.

### 2.6. Growth Experiment

Following hatch, only healthy, normally developed chicks with no visible physical abnormalities were considered eligible for the post-hatch growth trial. From this population, 300 chicks (48.23 ± 1.29 g initial live weight on average) were randomly selected and divided into five treatments with 6 replicates of 10 birds each. Straight-run commercial broiler chicks were used; therefore, sex was not determined at hatch, and randomization was expected to ensure a balanced sex distribution among treatments. The experiment was conducted at the Poultry Production Unit, Alexandria University, Alexandria (31.2001° N, 29.9187° E). Chicks were reared in a semi-close system building under uniform commercial conditions for 35 days, with *ad libitum* access to fresh water and a commercial diet “starter” containing 23% crude protein (CP) and 2950 kcal/kg of metabolizable energy (ME) during the brooding stage, while they were fed a grower–finisher diet containing 21% CP and 3150 kcal/kg ME during the fattening stage (Table 1). Body weight (BW), body weight gain (BWG), feed intake (FI), feed conversion ratio (FCR), and mortality rate were recorded weekly.

#### 2.6.1. Carcass Characteristics

At the end of the 35-day rearing period, 6 representative birds from each group were selected for slaughtering measurements. After slaughtering, birds were exsanguinated, then mechanically defeathered. The carcass was cut open and eviscerated, after which the empty carcass and the internal organs were separately weighed, and their relative weights were calculated.

#### 2.6.2. Blood Sampling

Blood samples were collected from 6 birds/treatment at 2 selected time points (1 and 35 d) for hematological and/or biochemical analysis, including protein profile, glucose, lipid profile, immune indices, growth hormones, liver and kidney function indicators, as well as oxidative stress markers.

##### Hematological and Biochemical Parameters

The red blood cell count (RBC) and hemoglobin (Hb), in addition to the white blood cell count (WBC) and their fractions (heterophil and lymphocyte percentages), were determined. While biochemical parameters included total protein, albumin, globulin, glucose, triglyceride, cholesterol levels, high-density lipoprotein (HDL), low-density lipoprotein (LDL), triiodothyronine (T_3_), thyroxin (T_4_), aspartate aminotransferase (AST), alanine aminotransferase (ALT), creatinine, uric acid, levels of immunoglobulin G (IgG) and immunoglobulin M (IgM), the activity of total antioxidant capacity (TAC), malondialdehyde (MDA), and glutathione peroxidase (GSH-Px) were commonly determined using the standard described methods [15].

### 2.7. Tissue Sampling and Analysis

Six samples of intestinal tissue for each treatment were collected at two time points: on the day of hatching (1 day of age) and at the end of the growth period (35 days of age) for morphological and histological analysis (e.g., intestine length, villus height, villus width, crypt depth, and villi surface area (VSA)).

### 2.8. Intestinal Bacterial Count

At the end of the growth period (35 days of age), intestinal content was collected from 6 representative birds for each treatment under sterilized conditions to determine the microbial count. Appropriate dilutions were plated on de Man, Rogosa and Sharpe (MRS) agar for *Lactobacillus* spp., MacConkey agar for *Escherichia coli*, and Xylose Lysine Deoxycholate (XLD) agar for *Salmonella* spp. Plates were incubated at 37 °C for 24–48 h under the recommended conditions for each microbial species. Microbial counts were expressed as log^10^ colony-forming unit (CFU)/g of intestinal digesta.

### 2.9. Statistical Analysis

Data were analyzed using a one-way ANOVA method with a significance level of *p* < 0.05. Data were subjected to analysis of variance using the General Linear Model (GLM) procedure of SAS software (version 9.4; SAS Institute Inc., Cary, NC, USA). Prior to analysis, the assumptions of normality and homogeneity of variance were evaluated using the Shapiro–Wilk and Levene’s tests, respectively. Tukey’s honestly significant difference (HSD) test was adopted for post hoc multiple comparisons. The results were considered significant at *p* ≤ 0.05. Additionally, orthogonal polynomial contrasts were utilized to evaluate the linear and quadratic responses of the measured parameters to the increasing levels of *in ovo* BGB injection.

## 3. Results

### 3.1. Hatchability and Chick Quality Traits at Hatch

The effects of *in ovo* BGB injection on hatchability traits and physical/morphometric characteristics of day-old broiler chicks are presented in Table 2. *In ovo* administration of BGB did not significantly alter egg weight or overall hatchability percentage across any of the experimental groups (*p* > 0.05). However, chick BW at hatch was significantly influenced by the treatments (*p* = 0.0484), exhibiting a strong linear increase (*p* = 0.0089) as the BGB level increased. Chicks hatched from eggs injected with 0.30% and 0.45% BGB achieved significantly higher BW compared to the untreated control group (*p* = 0.0484).

Chick quality, evaluated via the Pasgar score, was significantly modified by the treatments (*p* = 0.0296). A significant linear increase in Pasgar scores was observed (*p* = 0.0115), with the highest score recorded at the 0.30% injection level relative to both the control and saline-injected groups (*p* = 0.0296). Furthermore, chick length was significantly affected by the treatments (*p* = 0.0069), where all BGB-injected groups and the control group maintained statistically comparable lengths, while the saline group exhibited a notable reduction (*p* < 0.05). Conversely, shank length remained unaffected by any of the treatments (*p* > 0.05).

### 3.2. Serum Biochemical Parameters of Day-Old Broiler Chicks

The effects of *in ovo* injection of BGB at various concentrations (0.15%, 0.30%, and 0.45%) on the blood biochemical profiles of day-old broiler chicks are shown in Table 3. The experimental treatments yielded statistically significant variations (*p* < 0.05) across nearly all evaluated parameters, with the exception of serum albumin and T_4_ levels.

Serum total protein and globulin concentrations demonstrated a highly significant increase (*p* = 0.0005 and *p* = 0.0131, respectively) due to BGB injection. The highest total protein levels were achieved at 0.15% (5.24 g/dL) and 0.30% (5.26 g/dL) inclusion rates compared to the saline control (4.67 g/dL). Globulin values followed a similar pattern, peaking significantly at the 0.30% level (2.21 g/dL) relative to the saline group (1.64 g/dL). Conversely, serum albumin remained unaffected (*p* = 0.9527) across all treatments.

Active thyroid hormone (T_3_) concentrations increased sharply (*p* = 0.0001) in all BGB-treated groups (1.57 to 1.59 ng/mL) compared to both the un-injected control (1.41 ng/mL) and saline-injected control (1.36 ng/mL). This response exhibited a distinct linear trend (*p* = 0.0001). However, static T_4_ values exhibited no significant variations (*p* = 0.4041).

Serum glucose concentrations were reduced significantly (*p* = 0.0180) by BGB administration. Broiler chicks from the 0.15% BGB group displayed the lowest glucose levels (181.7 mg/dL), which were significantly lower than the saline control group (209.0 mg/dL).

Renal excretion metrics showed contrasting behaviors. Serum creatinine levels increased markedly (*p* = 0.0001) in higher treatment doses, nearly doubling in the 0.30% (0.52 mg/dL) and 0.45% (0.53 mg/dL) groups compared to the controls (0.28 to 0.29 mg/dL). In contrast, uric acid concentrations dropped significantly (*p* = 0.0499), reaching their lowest points in the 0.15% and 0.30% BGB groups (4.09 and 4.08 mg/dL, respectively) compared to the un-injected control (4.34 mg/dL).

Both serum AST and ALT activities were significantly modulated (*p* = 0.0460 and *p* = 0.0014, respectively). ALT activities declined notably in a linear fashion (*p* = 0.0015) as BGB was introduced, reaching a minimum at 0.30% BGB (20.00 U/L) before rising slightly at 0.45%. AST showed a similar decline, reaching its lowest value at 0.15% BGB (60.9 U/L), compared with the saline control (63.5 U/L).

### 3.3. Growth Performance Traits (1 to 35 Days)

The effects of *in ovo* BGB manipulation on long-term post-hatch growth performance parameters are presented in Table 4. Final BW (at 35 days) and BWG were significantly altered by the treatments (*p* = 0.0001). Both traits displayed highly significant linear and quadratic equations (*p* = 0.0001), establishing a distinct parabolic performance curve. Broilers from the 0.15% and 0.30% BGB injection groups achieved the highest final BW and weight gains, significantly outperforming the control, saline, and 0.45% BGB groups (*p* < 0.05).

Total feed consumption was significantly modified (*p* = 0.0047) due to a prominent quadratic response (*p* = 0.0004); FI increased up to the 0.30% dosage level but dropped significantly at the highest dose (0.45%). Crucially, the FCR was vastly optimized by the treatments (*p* = 0.0001), reflecting both linear (*p* = 0.0001) and quadratic (*p* = 0.0488) trends. All BGB-treated groups (0.15%, 0.30%, and 0.45%) yielded significantly lower, more efficient FCR values compared directly to the control and saline treatments (*p* < 0.05), with the 0.30% treatment recording the most favorable FCR value (1.49).

### 3.4. The Liver Functions, Kidney Functions, and Serum Lipid Profile of Broiler Chicks at 35 Days of Age

The effects of *in ovo* injection of different levels of BGB on the liver functions, kidney functions, and serum lipid profile of broiler chicks at 35 days of age are presented in Table 5. Significant differences were observed among the experimental groups (*p* = 0.0001). The *in ovo* injection of BGB significantly decreased AST activity compared to the control and saline groups. The reduction followed both a significant linear (*p* = 0.0001) and quadratic (*p* = 0.0001) trend, with the lowest enzyme activity recorded in the group treated with 0.30% BGB (45.0 U/L), followed by the 0.15% BGB group (52.7 U/L).

Serum ALT levels were not significantly affected by any of the BGB treatment levels, remaining stable across all experimental groups (*p* = 0.6698). No significant treatment effects were observed for serum creatinine levels (*p* = 0.2347), though a marginal downward linear trend was noted (*p* = 0.0693). There was a highly significant reduction in serum uric acid levels due to BGB treatments (*p* = 0.0001). All BGB-injected groups (0.15%, 0.30%, and 0.45%) showed significantly lower uric acid values (2.38, 2.42, and 2.43 mg/dL, respectively) compared to the control (2.97 mg/dL) and saline (2.72 mg/dL) groups. This reduction exhibited both highly significant linear (*p* = 0.0001) and quadratic (*p* = 0.0016) responses.

Serum cholesterol levels were significantly lowered by the BGB treatments (*p* = 0.0001). The lowest cholesterol concentration was achieved at the 0.30% BGB level (156.7 mg/dL), displaying strong linear (*p* = 0.0001) and quadratic (*p* = 0.0005) reductions compared to the control (183.3 mg/dL). BGB administration significantly decreased serum triglyceride levels (*p* = 0.0053). The lowest values were observed at BGB concentrations of 0.15% (77.0 mg/dL) and 0.30% (76.7 mg/dL), establishing a significant linear (*p* = 0.0094) and quadratic (*p* = 0.0032) effect. BGB treatments significantly increased serum HDL levels (*p* = 0.0086). The highest values were observed in the 0.15% (53.7 mg/dL) and 0.30% (54.0 mg/dL) BGB groups compared to the control group (47.2 mg/dL), confirming a significant linear increase (*p* = 0.0051). Serum LDL concentrations were significantly suppressed by BGB treatments (*p* = 0.0001), dropping from 118.6 mg/dL in the control group to a minimum of 87.3 mg/dL in the 0.30% BGB group. This reduction followed significant linear (*p* = 0.0001) and quadratic (*p* = 0.0002) patterns.

### 3.5. Hematological Traits, Immunological Parameters, and the Antioxidant Status of 35-Day-Old Broiler Chicks

The effects of *in ovo* BGB injection on hematological traits, immunological parameters, and the antioxidant status of 35-day-old broiler chicks are summarized in Table 6. The *in ovo* administration of BGB significantly altered hematological attributes (*p* < 0.05). RBC and Hb levels exhibited significant quadratic responses (*p* = 0.0006 and *p* = 0.0045, respectively) to increasing BGB levels. The highest RBC and Hb concentrations were recorded at the 0.15% and 0.30% BGB levels compared to both the control and saline groups. WBC increased linearly (*p* = 0.0376) and quadratically (*p* = 0.0098) with BGB treatment, peaking at the 0.30% level (35.1 · 10^3^/mm^3^). Furthermore, BGB injection led to a highly significant decrease in heterophil percentages (*p* = 0.0032) alongside a concurrent increase in lymphocyte percentages (*p* = 0.0024) across all inclusion levels compared to the control and saline cohorts.

Serum immunoglobulin concentrations were significantly modified by the treatments (*p* < 0.001). IgG levels responded in a linear (*p* = 0.0040) and quadratic (*p* = 0.0004) manner, reaching a maximum concentration (11.23 mg/mL) at 0.30% BGB. However, IgM values exhibited a strictly linear increase (*p* = 0.0001), maximizing at the highest treatment level of 0.45% BGB (5.34 mg/mL).

The serum antioxidant profile was markedly improved by BGB injection. TAC showed linear and significant upregulation (*p* = 0.0001), with all BGB groups (0.15%, 0.30%, and 0.45%) displaying statistically superior TAC values relative to the control and saline groups. GSH-PX activity also experienced a highly significant linear and quadratic boost (*p* = 0.0001 and *p* = 0.0011, respectively), scaling upwards from 41.2 U/mL in the control group to 66.0 U/mL in the 0.45% BGB group. Paralleling this enhanced antioxidant enzyme activity, concentrations of the lipid peroxidation biomarker MDA were significantly minimized (*p* = 0.0296), showing a distinct linear reduction (*p* = 0.0050) that bottomed out at 0.30% BGB (4.46 nmol/L).

### 3.6. Intestinal Morphometry and Histomorphology of Broiler Chicks at Day 35

The effects of *in ovo* BGB injection at different levels on the intestinal length and histomorphological parameters of broiler chicks at day 35 are presented in Table 7. The total length of the intestine was not significantly modified by any of the *in ovo* treatments (*p* = 0.6519), remaining statistically uniform across the control (197.0 cm), saline (201.0 cm), and BGB-treated groups (ranging from 204.0 to 212.0 cm).

There was a highly significant effect of the treatments on villi height (*p* = 0.0001). The *in ovo* injection of BGB at all tested levels (0.15%, 0.30%, and 0.45%) significantly increased villi height compared to both the untreated control and the saline-injected control groups (*p* < 0.05). This elevation demonstrated a strong linear (*p* = 0.0001) and quadratic (*p* = 0.0089) response, peaking at the 0.15% and 0.30% inclusion levels (1024 μm and 1017 μm, respectively). Villi width was significantly altered by the treatment (*p* = 0.0009). Broilers receiving 0.30% BGB exhibited the highest villi width (130.5 μm), which was significantly greater (*p* < 0.05) than that of the saline control group (106.3 μm) and the untreated control (115.3 μm). This development followed a distinct linear trajectory (*p* = 0.0015).

In contrast to the villi dimensions, crypt depth was not significantly influenced by the treatments (*p* = 0.9117), maintaining a steady baseline between 142.7 μm and 153.3 μm across all experimental groups. The villi-to-crypt depth ratio was profoundly enhanced by the treatments (*p* = 0.0020). All BGB treatment groups achieved significantly higher ratios compared to the control and saline groups (*p* < 0.05). This morphological index followed a highly significant linear pattern (*p* = 0.0004), with the maximum ratio observed at 0.30% BGB concentration (7.31). Conforming to the changes in villi height and width, the overall absorptive VSA expanded significantly due to the treatments (*p* = 0.0001). Broilers injected with BGB displayed significantly greater VSA values (0.39 to 0.42 μm^2^) relative to the control and saline groups (0.29 and 0.30 μm^2^, respectively) (*p* < 0.05). The change in VSA exhibited highly significant linear (*p* = 0.0001) and quadratic (*p* = 0.0142) trends.

### 3.7. Carcass Characteristics and Relative Organ Weights of 35-Day-Old Broiler Chicks

The effects of *in ovo* injection of varying levels of BGB on the carcass and internal organ relative weights of 35-day-old broiler chicks are presented in Table 8. The *in ovo* administration of BGB significantly influenced the quadratic response of dressing percentage (*p* = 0.0448), reaching its peak at the 0.30% BGB inclusion level (81.1%) compared to the control (77.7%) and saline (78.3%) groups. A strong trend toward a quadratic response was also observed for relative carcass weight (*p* = 0.0676), which reached a maximum value at 0.30% BGB inclusion (76.6%).

Conversely, the relative weights of the internal visceral organs and lymphoid tissues remained statistically unaffected by the *in ovo* BGB treatments (*p* > 0.05). Specifically, no significant differences were observed across treatment groups for the relative weights of the liver (*p* = 0.9869), gizzard (*p* = 0.3210), heart (*p* = 0.8542), and abdominal fat pad (*p* = 0.9944). Similarly, the relative weights of critical immune organs, including the spleen (*p* = 0.9970) and bursa of Fabricius (*p* = 0.6819), were not significantly altered by any concentration of BGB injection.

### 3.8. The Intestinal Microbial Counts

Regarding the intestinal microbiota profile on day 35 (Table 9), *in ovo* BGB injection significantly altered the intestinal microbial counts. For *Lactobacillus*, a significant linear increase was observed (*p* = 0.0300), with the population size rising from 8.78 log^10^ cfu/g in the control group to 9.58 log^10^ cfu/g in the 0.30% treatment group. Conversely, *Salmonella* populations were completely eliminated (0.00 log^10^ cfu/g, *p* = 0.0001) across all levels of BGB (0.15%, 0.30%, and 0.45%), exhibiting highly significant treatment, linear, and quadratic effects (*p* < 0.05). Similarly, *Escherichia coli* counts significantly decreased from 7.78 log^10^ cfu/g in the control group to 6.88, 6.83, and 6.62 log^10^ cfu/g in the 0.15%, 0.30%, and 0.45% BGB groups, respectively (*p* = 0.0141).

## 4. Discussion

The application of *in ovo* delivery technologies provides poultry embryos early access to critical nutrients and bioactive compounds, helping to modulate metabolic health well into the post-hatch growing phase. The current study investigates the effects of *in ovo* BGB administration at varying doses on the developmental and physiological responses of broiler chicks. The evaluation comprehensively assesses hatchability traits, early chick quality, and post-hatch growth performance over a 35-day rearing period. Additionally, carcass characteristics, the morphological and histological profiles of vital organs, intestinal microbiota populations, and systemic immunological and antioxidant indices are evaluated. According to the previous results, we indicate that *in ovo* BGB administration, particularly at the 0.30% level, serves as an effective developmental programming strategy that enhances intestinal maturation, metabolic efficiency, immune competence, and ultimately, carcass quality and long-term growth performance in broiler chickens.

### 4.1. Hatchability and Chick Quality Traits at Hatch

The lack of significant differences in hatchability percentages across all groups indicates that *in ovo* delivery of BGB up to a concentration of is safe and does not exert embryotoxic effects or obstruct the normal process of emergence (Table 2). Crucially, the significant linear escalation in day-old chick weights and improved Pasgar scores parallel findings where early administration of treatments provided supplementary dietary substrates. SCFAs like butyrate serve as foundational energy substrates for developing tissues, actively modulating intestinal development, nutrient transporter expression, and systemic immunity during the critical late-incubation embryonic phase [7]. Hence, in this study, BGB served as an immediate, high-efficiency energy source. This extra metabolic fuel helps the fully formed chick execute the energetic demands of pipping. Furthermore, the enhancement of chick physical measurements (Pasgar score and body length) indicates that supplying organic acids during late incubation bridges the typical transition window nutrient deficit and positively shapes the early metabolic framework of newly hatched broiler chicks [1,5,13].

### 4.2. Serum Biochemical Parameters of Day-Old Broiler Chicks

The highly significant increase in serum total protein and globulin concentrations, peaking at the 0.15% and 0.30% BGB inclusion rates, without an accompanying shift in albumin levels, strongly indicates an enhancement in protein anabolic pathways and humoral immunity (Table 3). Globulins constitute the foundational block of immunoglobulins (antibodies) in avian species. SCFAs like butyrate are well-documented to stimulate the proliferation and differentiation of intestinal epithelial cells and secondary lymphoid tissues [7,16]. Because maternal antibodies (IgY) are actively transferred from the yolk to embryonic circulation during the final days of incubation, the surge in globulins suggests that BGB optimizes this transfer or stimulates early endogenous immune programming without inducing systemic inflammation, as evidenced by stable albumin levels [17,18].

The sharp, linear increase in active T_3_ concentrations across all BGB groups, paired with static T_4_ levels, highlights direct metabolic acceleration. In avian physiology, T_3_ is the primary driver of hatching muscle glycogenolysis and lipolysis. This hormonal shift provides the physiological blueprint for the physical vigor required to execute internal and external pipping [19,20].

The significant reduction in serum glucose levels, particularly at the 0.15% BGB rate, initially seems counterintuitive given the hyper-metabolic state implied by elevated T_3_. However, this is a classic hallmark of rapid cellular uptake and energy shifts. When cells use external butyrate as a fuel source instead of glucose, it spares glucose for other vital body tissues [21]. The drop in circulating glucose may indicate that instead of maintaining high, unutilized blood glucose levels, the embryos are driving glucose into glycolysis and glycogen replenishment within the hatching muscles. This prevents the rapid depletion of glycogen stores during the anaerobic strains of pipping, effectively bridging the typical nutrient deficit of the transition window [22,23].

Additionally, the robust protein synthesis is heavily supported by the profound increase in serum T_3_ levels observed in all BGB-treated chicks. Thyroid hormones, especially T_3_, act as primary metabolic pacemakers that govern embryonic growth, heat production, and tissue differentiation in avian species. The sharp, linear expansion of T_3_ indicates that embryonic *in ovo* exposure to butyrate stimulates the peripheral monodeiodination of T_4_ to active T_3_, accelerating metabolic development to ready the chick for hatching. This hyper-metabolic state accounts for the lower serum glucose levels observed in BGB groups; elevated circulating T_3_ rapidly drives cellular glycolysis and tissue glucose uptake to meet the intense energy demands of late-stage embryo hatching [19,24,25].

The dramatic increase in serum creatinine at 0.30% and 0.45% BGB levels warrants careful physiological investigation. Creatinine is a byproduct of phosphocreatine breakdown in skeletal muscle tissues, and its systemic rise can be attributed to advanced skeletal muscle mass development [26,27]. This increase may stem from enhanced skeletal muscle development or a higher metabolic rate during pipping movements stimulated by elevated T_3_ levels [27,28,29]. The contrasting behavior between renal excretion metrics, where creatinine nearly doubled at higher doses while uric acid dropped significantly, reveals changes in skeletal muscle dynamics and nitrogen conservation. A drop in uric acid indicates that amino acids are being spared from oxidation and redirected toward tissue synthesis, a concept directly validated by the simultaneous rise in serum total proteins [28,30].

The linear decline in ALT and AST activities, hitting their nadir between 0.15% and 0.30% BGB, suggests a protective, stabilizing effect on hepatic tissue. ALT and AST are intracellular enzymes utilized to identify hepatic integrity. BGB components act as an energy source that stabilizes cellular membranes and downregulates oxidative stress markers [31,32,33]. The reduction in these enzymes confirms that BGB maintains hepatocyte integrity, protecting the liver as it manages the intense metabolic transformations of hatching.

Consequently, it can be inferred that injecting 0.15% to 0.30% BGB *in ovo* maximizes metabolic and immunological parameters in day-old chicks by enhancing active thyroid hormone secretion, accelerating protein synthesis, and shielding hepatic health.

### 4.3. Growth Performance Traits (1 to 35 Days)

The occurrence of highly significant linear and quadratic trends for final BW and BWG reveals a biological optimal threshold, peaking between the 0.15% and 0.30% BGB inclusion rates (Table 4). The drastic decline in final BW at the 0.45% dosage level indicates a metabolic ceiling. Excessively high concentrations of SCFAs inside the amniotic fluid or embryonic gut can disrupt the osmotic balance or trigger localized chemical irritation in the developing intestinal mucosa. Furthermore, high concentrations of systemic butyrate can negatively affect appetite stimulation, explaining the corresponding drop in total feed consumption observed at the 0.45% level [34]. Excessive concentrations of free volatile organic acids in embryonic fluids may also delay embryonic yolk sac absorption or mildly downregulate FI pathways post-hatch [1]. Exogenous butyrate serves as the primary, preferred energy substrate for intestinal colonocytes and mucosal development [31]. Additionally, *in ovo* provisioning of butyrate dramatically increases jejunal and ileal villus height, crypt depth, and total mucosal surface area at hatch, which has a profound impact on increasing body weight [23,35].

### 4.4. The Liver Functions, Kidney Functions, and Serum Lipid Profile of Broiler Chicks at 35 Days of Age

The highly significant linear and quadratic reductions in serum AST activity, hitting their lowest point at the 0.30% BGB level (45.0 U/L), combined with completely stable ALT levels, indicate enhanced, long-term hepatocyte health and structural integrity (Table 5). Butyrate derivatives act as cellular signaling molecules that reduce hepatic oxidative stress by upregulating major antioxidant networks, including glutathione peroxidase and superoxide dismutase [36,37]. The suppressed AST baseline at day 35 proves that early *in ovo* BGB exposure creates a permanent, protective adaptation in liver cell membrane architecture. This guards hepatocytes against the lipotoxicity and cellular leaking normally caused by modern, high-nutrient commercial diets [11].

In avian species, uric acid is the primary end-product of purine and amino acid catabolism. Because blood creatinine remained baseline stable across all groups, the drop in uric acid is not due to renal failure or impaired elimination. Instead, it indicates a highly efficient metabolic shift where the bird’s system wastes less dietary protein as nitrogenous waste. This lifelong reduction in uric acid perfectly aligns with the superior final BW and optimized 1.49 FCR seen at the 0.30% level, indicating that BGB can shift the broiler’s core metabolic blueprint toward lean meat accretion and improved nitrogen balance [38].

Administering BGB can effectively reduce the synthesis of circulating triglycerides and very-low-density lipoproteins (VLDL, the precursor to LDL) [39,40]. The simultaneous drop in LDL (“bad cholesterol”) and the surge in HDL (“good cholesterol”) indicate that BGB can upregulate reverse cholesterol transport mechanisms. HDL acts to scavenge peripheral tissue cholesterol and transport it back to the liver for bile acid conversion and subsequent excretion. This metabolic shift reduces abdominal and visceral fat deposition—which often compromises carcass quality in modern strains—and instead directs energy into building valuable pectoral and skeletal muscle mass [34,41].

Ultimately, the biochemical data at Day 35 demonstrate that the physiological and metabolic programming initiated by *in ovo* administration of BGB persists as a lifetime structural change, enduring long after the initial embryonic intervention. By day 35, the experimental treatments have altered the metabolic trajectory of market-age broilers, creating a more efficient hepatic framework and establishing a highly favorable serum lipid profile.

### 4.5. Hematological Traits, Immunological Parameters, and the Antioxidant Status of 35-Day-Old Broiler Chicks

The hematological, immunological, and antioxidant profiles at day 35 provide definitive evidence that *in ovo* administration of BGB induces a profound, lifelong physiological restructuring in broiler chickens. Intervening during the late embryonic phase creates an adaptive momentum that persists across the entire 35-day production cycle, manifesting as enhanced oxygen-carrying capacity, heightened systemic immunity, and superior systemic antioxidant defense mechanisms. The widespread emergence of significant linear and quadratic trends, consistently peaking within the 0.15% to 0.30% therapeutic window, uncovers the exact biological mechanisms behind how this SCFA derivative permanently optimizes broiler health (Table 6).

The highly significant quadratic responses in RBCs and Hb concentrations, which peaked at the 0.15% and 0.30% BGB inclusion levels, point toward an upgraded hematopoietic infrastructure. Late-term embryonic development and the subsequent hatching process expose chicks to severe hypoxia and high physical strain. BGB serves as a highly efficient energetic substrate that promotes cell proliferation [42]. Expanding the baseline circulating erythrocyte pool and hemoglobin capacity at day 35 equips broilers with superior tissue oxygenation capabilities. This enhanced oxygen transport network directly sustains the metabolic demands of rapid skeletal muscle hypertrophy, helping explain the increased final BW and optimized FCR seen at the 0.30% inclusion level [43,44].

The notable linear and quadratic expansion of total WBC, peaking at 0.30% BGB, combined with a significant reduction in heterophil percentages and a concurrent rise in lymphocytes, indicates a powerful shift toward systemic immune competence and stress resilience [45]. The downward shift in heterophils alongside an increase in lymphocytes across all BGB groups proves that early *in ovo* exposure permanently lowers the baseline physiological stress of the broiler. This leukocyte polarization suggests that BGB stabilizes homeostatic regulation, allowing the birds to navigate the metabolic pressures of commercial rearing without triggering harmful, energy-draining inflammatory responses [2,46].

The substantial upregulation of serum immunoglobulins, where IgG (the avian counterpart to mammalian IgG, functionally termed IgY) peaked quadratically at 0.30% BGB (11.23 mg/mL), and IgM increased linearly up to 0.45%, demonstrates a highly active humoral immune framework. The vast majority of a chicken’s immune programming occurs within the gut-associated lymphoid tissue (GALT) and the bursa of Fabricius during late incubation and the first week post-hatch. Because BGB provides the preferred fuel for early enterocyte development, it directly accelerates the morphological maturation of the intestinal mucosa and its underlying lymphoid structures [2,35]. Butyrate activates G-protein-coupled receptors (such as *GPR41* and *GPR43*) on immune cells, signaling a cascade that promotes B-cell differentiation and plasma cell immunoglobulin production [7]. Maximizing IgG—the primary antibody responsible for systemic, long-term secondary immune defense—at the 0.30% level explains how these birds maintain superior health, translating robust immune protection directly into enhanced longitudinal growth performance.

The sweeping, linear upregulation of TAC and GSH-Px activity, running parallel to a significant linear drop in the lipid peroxidation biomarker MDA, confirms that BGB provides lifelong cellular protection. Rapid growth in modern broiler strains generates immense quantities of intracellular free radicals (reactive oxygen species, ROS), which can easily damage cell membranes, leading to tissue degradation marked by elevated liver enzymes like AST. GSH-Px is the body’s primary enzymatic weapon for neutralizing these damaging peroxides. Exogenous butyrate derivatives alter cell signaling pathways to upregulate nuclear factor erythroid 2-related factor 2 (Nrf2), the master transcription factor governing antioxidant enzyme expression [31]. The fact that GSH-Px scaled up to 66.0 U/mL while MDA bottomed out at 4.46 nmol/L at the 0.30% level demonstrates a highly efficient system. The birds’ cells are completely protected from lipid peroxidation, preserving structural cell membranes throughout the body. This systemic stabilization prevents intracellular enzyme leakage (fully validating the depressed day 35 AST levels), ensuring that nutrients are strictly directed toward growth.

### 4.6. Intestinal Morphometry and Histomorphology of Broiler Chicks at Day 35

The intestinal morphometry and histomorphological data at day 35 provide a definitive anatomical explanation for the superior growth performance and optimized FCR observed in the BGB-treated broilers (Table 7). Intervening *in ovo* during late embryogenesis establishes a lifelong structural legacy within the gastrointestinal tract. Rather than increasing the gross macroscopic length of the intestine, early exposure to BGB drives microscopic architectural remodeling, expanding the functional mucosal boundary to maximize nutrient assimilation throughout the broiler’s lifespan. The prominent linear and quadratic responses—culminating around the 0.15% to 0.30% inclusion window—reveal the exact cellular and biophysical mechanisms behind this permanent gut optimization.

The lack of significant variation in total intestinal length (ranging uniformly from 197.0 to 218.0 cm) alongside the drastic changes in microscopic mucosal dimensions highlights a fundamental principle of developmental programming: BGB can act as a regulator of cellular differentiation and tissue quality rather than raw organ elongation. Elongating the physical gut loop requires substantial visceral space and increases metabolic maintenance costs, as the gastrointestinal tract is one of the most energy-demanding organ systems in poultry [23]. Instead of forcing macroscopic expansion, *in ovo* BGB delivery directs cellular resources toward multiplying and expanding individual functional units—the villi—within the fixed structural framework of the gut loop. This strategy allows the bird to expand its digestive capacity without increasing tissue maintenance burdens [3].

The highly significant linear and quadratic increases in villi height (peaking at 1024 μm in the 0.15% group) and villi width (peaking at 130.5 μm in the 0.30% group) demonstrate a massive structural expansion of the gut mucosa. Butyrate is the preferred and highly efficient energy substrate for the rapidly proliferating intestinal epithelium [6,31]. During the late incubation phase and early post-hatch transition window, the embryonic gut undergoes a massive wave of enterocyte proliferation. Supplying exogenous butyrate derivatives *in ovo* directly accelerates enterocyte mitotic activity along the villus axis [35]. This early energy boost protects the newly hatched chick from the typical mucosal atrophy and villus shortening caused by the post-hatch fasting window, ensuring that the villi remain tall and wide through day 35.

The completely static baseline of crypt depth (remaining non-significant at *p* = 0.9117 across all groups) combined with enhanced villi height yields a highly significant, linear enhancement of the villi-to-crypt depth (V:C) ratio, peaking at 7.31 in the 0.30% BGB group. It is well known that the V:C ratio is the definitive histomorphological index for estimating intestinal health, tissue turnover costs, and absorptive efficiency [47]. The steady crypt depth observed here proves that the taller and wider villi in the BGB groups explain the highly efficient FCR (1.49) achieved at the 0.30% BGB level.

The ultimate phenotypic expression of these micro-structural alterations is the dramatic expansion of the calculated absorptive VSA, which surged from 0.29 to 0.30 μm^2^ in the controls to a maximum of 0.42 μm^2^ in the BGB groups. In intestinal biophysics, fluid dynamics dictate that nutrient absorption is directly proportional to the total contact area between digested luminal chyme and the brush border membrane. By simultaneously driving up villi height and width through both linear and quadratic pathways, BGB expands the physical contact zone for nutrient assimilation [48]. This permanent expansion of the VSA means that for every centimeter of digesta passing through the intestinal lumen, a BGB-treated broiler can extract and transport amino acids, lipids, and carbohydrates at a significantly higher rate than untreated controls. This optimized digestive infrastructure explains how the birds maintain a superior nitrogen balance (lowered day 35 uric acid) and sustain accelerated growth performance over the entire 35-day production cycle.

### 4.7. Carcass Characteristics and Relative Organ Weights of 35-Day-Old Broiler Chicks

The evaluation of carcass yields and relative organ weights at 35 days of age outlines the anatomical and structural changes driven by the developmental programming of *in ovo* BGB injection. The appearance of a significant quadratic response in dressing percentage alongside a robust quadratic trend in relative carcass weight—both maximizing precisely at the 0.30% BGB threshold—proves that early-life embryonic interventions actively reshape market-age yield (Table 8). Conversely, the completely static relative weights of visceral organs and lymphoid tissues indicate that this intervention specifically directs nutrients into edible skeletal muscle mass rather than internal organ hypertrophy.

Late-term embryonic development (the pre-hatch phase) is a critical period for establishing a chick’s lifelong muscle potential. The significant quadratic peak in dressing percentage at the 0.30% BGB inclusion level (81.1%), compared to the control (77.7%) and saline (78.3%) cohorts, demonstrates a highly efficient translation of metabolic energy into skeletal meat yield, as butyrate acts as an efficient energy substrate, generating the necessary cellular energy to support early myoblast proliferation [49,50,51,52]. The increase in early muscle fiber capacity provides the physiological foundation for the linear increase in carcass weight and superior dressing percentage seen at market age.

The absolute lack of statistical variation (*p* > 0.05) in the relative weights of the liver, gizzard, and heart indicates that early BGB manipulation does not cause physical enlargement or visceral tissue distortion. In commercial poultry production, an increase in the relative weight of visceral organs (such as the liver or gastrointestinal segments) often represents a metabolic drawback. Visceral organs are highly energy-expensive tissues, accounting for a massive proportion of whole-body oxygen consumption and protein turnover relative to their mass [23]. The fact that the liver, gizzard, and heart remained proportional across all groups confirms that BGB improves functional efficiency rather than tissue mass. This aligns perfectly with the lower circulating liver enzymes (AST) and lower blood uric acid observed at 35 days. The internal organs are biochemically protected and metabolically streamlined, allowing the bird to safely route dietary nutrients away from visceral maintenance and directly toward muscle tissue deposition [53]. The completely stable relative weight of the abdominal fat pad (*p* = 0.9944), alongside the major drops in serum cholesterol, triglycerides, and LDL, confirms that *in ovo* BGB shifts energy processing pathways away from fat storage. SCFAs like butyrate reduce the activity of fat-synthesizing enzymes (such as fatty acid synthase) in the avian liver while boosting fatty acid breakdown in peripheral tissues [54]. Rather than depositing excess energy as an abdominal fat pad, BGB-programmed broilers channel those lipid substrates into muscle development or use them to power daily growth, resulting in a cleaner, higher-yielding carcass at slaughter [55].

### 4.8. The Intestinal Microbial Counts

The intestinal microbiota data at day 35 demonstrate that *in ovo* administration of a BGB exerts a profound, long-term regulatory control over the intestinal ecosystem of broiler chickens. The significant linear proliferation of beneficial lactic acid bacteria (*Lactobacillus*), the absolute eradication of zoonotic *Salmonella*, and the substantial downregulation of pathogenic *Escherichia coli* reveal that this single embryonic intervention successfully guides the structural assembly of the mature gut microbiome (Table 9). The persisting microbial shifts through day 35 provide a clear biological mechanism for optimized growth, enhanced immune competence, and systemic health observed across the production cycle.

The highly significant linear increase in intestinal *Lactobacillus* populations—peaking at the 0.30% BGB inclusion level—highlights the establishment of a robust, self-sustaining “eubiotic” (optimally balanced) intestinal microflora. *In ovo*-injected butyrate glycerides serve as an early, highly bioavailable energy substrate that accelerates enterocyte development and mucosal barrier assembly during late incubation [4,35]. This early gastrointestinal head start optimizes crypt-villus architecture, creating an ideal ecological niche for fast-growing saccharolytic bacteria. As these beneficial *Lactobacillus* species proliferate, they ferment dietary carbohydrates to produce lactic acid and secondary SCFAs, lowering the local digesta pH inside the cecal pouches [6,23]. Proliferating *lactobacilli* outcompete environmental pathogens for mucosal attachment sites and essential nutrients. This highly efficient biological barrier prevents opportunistic pathogens from colonizing the intestinal wall, a protective phenomenon natively supported by modern poultry microbiomics [7,56].

The absolute eradication of *Salmonella* species (0.00 log^10^ cfu/g) across all BGB treatment levels, paired with the sharp reduction in *Escherichia coli* counts, represents a major milestone for both bird health and food safety. Acidophilic, beneficial bacteria like *Lactobacillus* thrive in slightly acidic environments, but enteropathogens like *Salmonella* and *E. coli* are highly sensitive to pH drops. In their undissociated form, SCFAs like butyrate pass easily through the semi-permeable lipid membranes of Gram-negative bacterial cells. Once inside the neutral cytoplasm, the acid dissociates, releasing hydrogen ions (H^+^) that drop the internal pH. This forced acidification exhausts the bacteria’s cellular energy reserves as it pumps out excess protons, ultimately causing metabolic collapse and cell death [48,57]. Beyond direct cellular toxicity, butyrate acts as a targeted molecular signaling agent. At sub-lethal concentrations, butyrate directly downregulates the expression of *Salmonella* pathogenicity island 1 (SPI-1) genes, effectively blinding the pathogen and preventing it from invading the host’s intestinal epithelial cells [58]. This multi-layered antimicrobial attack explains the complete clearance of *Salmonella* and the significant linear reduction in *E. coli* down to 6.62 log^10^ cfu/g.

## 5. Conclusions

*In ovo* administration of BGB significantly enhances broiler chick development without compromising hatchability, establishing a distinct parabolic performance curve that peaks at the 0.30% inclusion level. This optimal dosage maximizes 35-day BW, feed efficiency (FCR of 1.49), and dressing percentage (81.1%) by profoundly improving intestinal morphometry (villi-to-crypt ratio of 7.31) and expanding the absorptive surface area. Furthermore, the 0.30% treatment fosters a healthier intestinal microbiome by significantly increasing beneficial *Lactobacillus* while completely eliminating *Salmonella*, alongside boosting systemic immunity (IgG and IgM), enhancing antioxidant defense (TAC and GSH-PX), and optimizing the lipid profile without inducing long-term hepatic or renal toxicity. Therefore, it is highly recommended to implement *in ovo* BGB injection at a concentration of 0.30% in commercial poultry production to achieve the ideal balance of maximized growth, robust gut health, and superior disease resistance.

## Figures and Tables

**Table 1:** Composition and chemical analysis of commercial starter and grower-finisher diets supplied to broilers throughout the experimental growth periods.

Ingredients (kg)	Experimental Diets	
	Starter Period (1–20 d)	Grower–Finisher Period (21–35 d)
Yellow corn	555	600
Soybean meal 46%	344	290
Corn gluten meal 60%	40	42
Soybean oil	17	25
Mono calcium phosphate	15	14
Limestone	16	16
Sodium chloride (Salt)	3.8	3.8
Vit. & minerals premix *	3.0	3.0
Choline chloride	1.0	1.0
DL-Methionine	2.7	2.5
L-Lysine Hcl	2.5	2.7
Total	1000	1000
Chemical analysis		
Crude protein, %	23.00	21.00
Metabolizable energy, K.cal/kg	2950	3150
Ether extract, %	4.30	5.20
Crude fiber, %	2.30	2.10
Calcium, %	1.00	0.96
Available phosphorus, %	0.50	0.48
Lysine, %	1.50	1.40
Methionine, %	0.69	0.64

* Premix supplied per 3 kg of diet: Vit. A, 12,000,000 IU; Vit. E, 10,000 mg; Vit. B1, 1000 mg; Vit. B2, 5000 mg; Vit. B6, 1500 mg; Vit. B12, 10 mg; Niacin, 30,000 mg; Pantothenic acid, 15,000 mg; Vit. K, 2000 mg; Vit. D3, 2,200,000 IU; Biotin, 50 mg; Folic acid, 1000 mg; Cu, 4000 mg; I, 2000 mg; Fe, 30,000 mg; Mn, 60,000 mg; Zn, 50,000 mg; Se, 400 mg; and Co, 100 mg.

**Table 2:** Effect of *in ovo* butyrate glyceride blend (BGB) injection on hatchability and chick quality traits of day-old broiler chicks.

Item	Control	Saline	BGB, %			SEM	*p*-Value		
			0.15	0.30	0.45		T	L	Q
Egg, g	69.2	69.8	68.9	69.3	69.3	0.519	0.3879	0.6223	0.6060
Chick, g	48.1 ^b^	48.9 ^ab^	48.8 ^ab^	49.8 ^a^	49.6 ^a^	0.382	0.0484	0.0089	0.5010
Hatchability, %	98.6	98.9	98.7	98.7	98.7	0.595	0.9983	0.9545	0.8920
Pasgar score	9.53 ^b^	9.50 ^b^	9.73 ^ab^	9.83 ^a^	9.70 ^ab^	0.068	0.0296	0.0115	0.2214
Chick Length, cm	19.93 ^a^	19.43 ^b^	20.03 ^a^	20.20 ^a^	19.93 ^a^	0.111	0.0069	0.0531	0.9374
Shank length, cm	5.00	4.97	4.97	5.00	4.97	0.077	0.7368	0.8945	0.4395

^a,b.^ Means within the same row with different letters are significantly different (*p* < 0.05). SEM, standard error of mean; T, treatment; L, linear; Q, quadratic.

**Table 3:** Effect of *in ovo* butyrate glyceride blend (BGB) injection at different levels on some biochemical parameters of day-old broiler chicks.

Items	Control	Saline	BGB, %			SEM	*p*-Value		
			0.15	0.30	0.45		T	L	Q
Total protein (g/dL)	4.92 ^b^	4.67 ^c^	5.24 ^a^	5.26 ^a^	5.12 ^ab^	0.068	0.0005	0.0011	0.2347
Albumin (g/dL)	3.09	3.03	3.07	3.05	3.10	0.074	0.9527	0.8451	0.5742
Globulin (g/dL)	1.84 ^bc^	1.64 ^c^	2.17 ^ab^	2.21 ^a^	2.02 ^ab^	0.101	0.0131	0.0157	0.2308
Glucose (mg/dL)	199.0 ^b^	209.0 ^a^	181.7 ^c^	186.3 ^c^	192.7 ^bc^	4.817	0.0180	0.0428	0.2011
T_3_ (ng/mL)	1.41 ^b^	1.36 ^b^	1.57 ^a^	1.59 ^a^	1.58 ^a^	0.022	0.0001	0.0001	0.1879
T_4_ (ng/mL)	6.22	6.21	6.43	6.43	6.35	0.100	0.4041	0.1693	0.3742
Creatinine (mg/dL)	0.29 ^b^	0.28 ^b^	0.31 ^b^	0.52 ^a^	0.53 ^a^	0.012	0.0001	0.0001	0.0001
Uric acid (mg/dL)	4.34 ^a^	4.19 ^ab^	4.09 ^b^	4.08 ^b^	4.17 ^ab^	0.056	0.0499	0.0279	0.0231
AST (U/L)	63.2 ^ab^	63.5 ^a^	60.9 ^c^	61.1 ^bc^	62.4 ^ab^	0.632	0.0460	0.0810	0.0679
ALT (U/L)	24.7 ^a^	24.0 ^ab^	20.7 ^cd^	20.00 ^d^	22.3 ^bc^	0.632	0.0014	0.0015	0.0044

^a,b,c,d.^ Means within the same row with different letters are significantly different (*p* < 0.05). SEM, standard error of mean; T, treatment; L, linear; Q, quadratic; T_3_, triiodothyronine; T_4_, thyroxine. AST, aspartate aminotransferase; ALT, alanine aminotransferase.

**Table 4:** Effect of *in ovo* butyrate glyceride blend (BGB) injection at different levels on performance traits of broiler chicks.

Item	Control	Saline	BGB, %			SEM	*p*-Value		
			0.15	0.30	0.45		T	L	Q
Initial body weight (g)	48.1 ^b^	48.9 ^ab^	48.8 ^ab^	49.8 ^a^	49.6 ^a^	0.382	0.0484	0.0089	0.5010
Final body weight (g)	2094 ^c^	2087 ^c^	2277 ^a^	2300 ^a^	2149 ^b^	19.06	0.0001	0.0001	0.0001
Body weight gain (g)	2046 ^c^	2038 ^c^	2228 ^a^	2251 ^a^	2100 ^b^	19.07	0.0001	0.0001	0.0001
Feed consumption (g)	3305 ^ab^	3351 ^a^	3388 ^a^	3345 ^a^	3244 ^b^	28.14	0.0047	0.1444	0.0004
Feed conversion ratio (g: g)	1.63 ^a^	1.65 ^a^	1.52 ^b^	1.49 ^b^	1.55 ^b^	0.024	0.0001	0.0001	0.0488

^a,b,c.^ Means within the same row with different letters are significantly different (*p* < 0.05). SEM, standard error of mean; T, treatment; L, linear; Q, quadratic.

**Table 5:** Effect of *in ovo* butyrate glyceride blend (BGB) injection at different levels on liver/kidney functions and lipid profile of broiler chicks at the end of growth period (35 d).

Items	Control	Saline	BGB, %			SEM	*p*-Value		
			0.15	0.30	0.45		T	L	Q
AST (U/L)	74.7 ^a^	67.7 ^b^	52.7 ^d^	45.0 ^e^	59.3 ^c^	1.468	0.0001	0.0001	0.0001
ALT (U/L)	20.3	20.2	20.0	19.00	20.3	0.719	0.6698	0.6189	0.4392
Creatinine (mg/dL)	0.63	0.60	0.55	0.57	0.56	0.025	0.2347	0.0693	0.2475
Uric acid (mg/dL)	2.97 ^a^	2.72 ^b^	2.38 ^c^	2.42 ^c^	2.43 ^c^	0.058	0.0001	0.0001	0.0016
Cholesterol (mg/dL)	183.3 ^a^	175.7 ^b^	162.0 ^cd^	156.7 ^d^	167.7 ^c^	2.399	0.0001	0.0001	0.0005
Triglyceride (mg/dL)	88.0 ^a^	84.4 ^a^	77.0 ^bc^	76.7 ^c^	82.7 ^ab^	1.817	0.0053	0.0094	0.0032
HDL (mg/dL)	47.2 ^b^	46.8 ^b^	53.7 ^a^	54.0 ^a^	51.3 ^ab^	1.378	0.0086	0.0051	0.0550
LDL (mg/dL)	118.6 ^a^	111.9 ^a^	92.9 ^bc^	87.3 ^c^	99.8 ^b^	2.421	0.0001	0.0001	0.0002

^a,b,c,d,e.^ Means within the same row with different letters are significantly different (*p* < 0.05). SEM, standard error of mean; T, treatment; L, linear; Q, quadratic; AST, aspartate aminotransferase; ALT, alanine aminotransferase; HDL, high-density lipoprotein; LDL, low-density lipoprotein.

**Table 6:** Effect of *in ovo* butyrate glyceride blend (BGB) injection at different levels on immunological parameters and antioxidant status of broiler chicks at the end of growth period (35 d).

Items	Control	Saline	BGB, %			SEM	*p*-Value		
			0.15	0.30	0.45		T	L	Q
RBC (10^6^/mm^3^)	3.01 ^b^	3.02 ^b^	3.64 ^a^	3.53 ^a^	3.18 ^b^	0.079	0.0004	0.0068	0.0006
Hb (g/dL)	11.87 ^c^	12.07 ^c^	12.83 ^ab^	13.37 ^a^	12.33 ^bc^	0.198	0.0020	0.0051	0.0045
WBC (10^3^/mm^3^)	29.4 ^c^	30.2 ^c^	33.8 ^ab^	35.1 ^a^	30.9 ^bc^	1.032	0.0129	0.0376	0.0098
Heterophils (%)	24.3 ^a^	23.5 ^a^	17.67 ^b^	17.33 ^b^	19.33 ^b^	1.095	0.0032	0.0009	0.0217
Lymphocytes (%)	70.7 ^b^	71.5 ^b^	77.3 ^a^	77.7 ^a^	75.7 ^a^	1.095	0.0024	0.0009	0.0217
IgG (mg/mL)	9.91 ^c^	9.83 ^c^	10.75 ^b^	11.23 ^a^	10.02 ^c^	0.137	0.0001	0.0040	0.0004
IgM (mg/mL)	4.47 ^c^	4.52 ^c^	4.97 ^b^	5.26 ^ab^	5.34 ^a^	0.103	0.0003	0.0001	0.7881
MDA (nmol/L)	4.92 ^ab^	4.96 ^a^	4.73 ^abc^	4.46 ^c^	4.58 ^bc^	0.105	0.0296	0.0050	0.7660
TAC (μmol/L)	1.73 ^b^	1.65 ^b^	1.97 ^a^	2.10 ^a^	2.00 ^a^	0.046	0.0002	0.0001	0.2219
GSH-PX (U/mL)	41.2 ^d^	51.4 ^c^	60.7 ^b^	63.7 ^ab^	66.0 ^a^	1.313	0.0001	0.0001	0.0011

^a,b,c,d.^ Means within the same row with different letters are significantly different (*p* < 0.05). SEM, standard error of mean; T, treatment; L, linear; Q, quadratic; RBC, red blood cell count; Hb, hemoglobin; WBC, white blood cell count; IgG, immunoglobulin G; IgM, immunoglobulin M; MDA, malondialdehyde; TAC, total antioxidant capacity; GSH-PX, glutathione peroxidase.

**Table 7:** Effect of *in ovo* butyrate glyceride blend (BGB) injection at different levels on intestine length and histological parameters of broiler chicks at the end of the experimental growth period (35 d).

Items	Control	Saline	BGB, %			SEM	*p*-Value		
			0.15	0.30	0.45		T	L	Q
Intestine length (cm)	197.0	201.0	212.0	218.0	204.0	10.71	0.6519	0.3815	0.3303
Villi height (μm)	803.5 ^b^	793.2 ^b^	1024 ^a^	1017 ^a^	997 ^a^	24.13	0.0001	0.0001	0.0089
Villi width (μm)	115.3 ^bc^	106.3 ^c^	126.8 ^ab^	130.5 ^a^	124.8 ^ab^	3.819	0.0009	0.0015	0.4834
Crypt depth (μm)	153.3	147.7	146.7	142.7	147.0	7.762	0.9117	0.4784	0.5636
Villi/Crypt ratio	5.27 ^b^	5.41 ^b^	7.04 ^a^	7.31 ^a^	6.98 ^a^	0.409	0.0020	0.0004	0.1448
VSA (μm^2^)	0.29 ^b^	0.30 ^b^	0.41 ^a^	0.42 ^a^	0.39 ^a^	0.017	0.0001	0.0001	0.0142

^a,b,c.^ Means within the same row with different letters are significantly different (*p* < 0.05). SEM, standard error of mean; T, treatment; L, linear; Q, quadratic; VSA, villi surface area.

**Table 8:** Effect of *in ovo* butyrate glyceride blend (BGB) injection at different levels on carcass and internal organs relative weights (%) of broiler chicks at the end of the experimental growth period (35 d).

Items	Control	Saline	BGB, %			SEM	*p*-Value		
			0.15	0.30	0.45		T	L	Q
Carcass	73.4	74.0	76.1	76.6	74.4	0.935	0.1434	0.1437	0.0676
Dressing	77.7	78.3	80.6	81.1	78.8	0.895	0.0827	0.0958	0.0448
Liver	2.65	2.62	2.82	2.73	2.69	0.272	0.9869	0.8295	0.7717
Gizzard	1.16	1.18	1.29	1.31	1.29	0.060	0.3210	0.0701	0.4481
Heart	0.44	0.43	0.45	0.42	0.42	0.026	0.8542	0.4573	0.6644
Abdominal fat	1.05	1.04	1.02	1.00	1.02	0.096	0.9944	0.7249	0.8496
Spleen	0.17	0.16	0.17	0.17	0.17	0.030	0.9970	0.8382	0.9541
Bursa	0.10	0.10	0.11	0.11	0.11	0.009	0.6819	0.2659	0.5634

SEM, standard error of mean; T, treatment; L, linear; Q, quadratic.

**Table 9:** Effect of *in ovo* butyrate glyceride blend (BGB) injection at different levels on intestinal microbiota count (log^10^ cfu/g) of broiler chicks at the end of the growth period (35 d).

Items	Control	Saline	BGB, %			SEM	*p*-Value		
			0.15	0.30	0.45		T	L	Q
*Lactobacillus*	8.78	8.93	9.47	9.58	9.40	0.235	0.1368	0.0300	0.2452
*Salmonella*	3.49 ^b^	3.52 ^a^	0.00 ^c^	0.00 ^c^	0.00 ^c^	0.004	0.0001	0.0001	0.0001
*E. coli*	7.78 ^a^	7.63 ^ab^	6.88 ^b^	6.83 ^b^	6.62 ^b^	0.324	0.0141	0.0127	0.6386

^a,b,c.^ Means within the same row with different letters are significantly different (*p* < 0.05). SEM, standard error of mean; T, treatment; L, linear; Q, quadratic.

## Data Availability

The data can be made available from the corresponding author upon reasonable request.
